# Pharmacokinetic and Pharmacodynamic Considerations in Relation to Calcineurin Usage in Elderly Kidney Transplant Recipients

**DOI:** 10.3389/fphar.2021.635165

**Published:** 2021-04-12

**Authors:** Amelia R. Cossart, Nicole M. Isbel, Carla Scuderi, Scott B. Campbell, Christine E. Staatz

**Affiliations:** ^1^ School of Pharmacy, University of Queensland, Brisbane, QLD, Australia; ^2^ Department of Nephrology, University of Queensland at the Princess Alexandra Hospital, Brisbane, QLD, Australia; ^3^ Kidney Health Service, Royal Brisbane and Women’s Hospital, Brisbane, QLD, Australia

**Keywords:** kidney, transplantation, calcineurin inbibitors, elderly, immunosuppression, pharmacokinetics, pharmacodynamics, therapeutic drug monitoring

## Abstract

This review summarizes how possible age-related changes in tacrolimus and cyclosporine pharmacokinetics and pharmacodynamics may influence drug dosing and monitoring in the elderly, and highlights how micro-sampling may be useful in this cohort in the future. Advancing biological age leads to physiological changes that can affect drug absorption, distribution, metabolism and excretion, as well as immune system responsiveness. Some studies have shown that elderly recipients may have higher dose-adjusted exposure and/or lower clearance of the calcineurin inhibitors, suggesting that doses may need to be lowered in elderly recipients. Only one study has examined how aging effects drug target enzyme activity and demonstrated that age does not correlate with the calcineurin inhibitor half-maximal inhibitory concentration. Several studies have shown elderly kidney transplant recipients have increased risk of both morbidity and mortality, compared to younger adults due to increased susceptibility to immunosuppressant side effects, particularly cardiovascular disease, infection and malignancy. Current immunosuppressant dosing and monitoring protocols often make no adjustments for age. Lower maintenance immunosuppressant targets in elderly recipients may decrease patient susceptibility to drug side effects, however, further studies are required and appropriate targets need to be established. Blood draw by micro-sampling may be useful for drug monitoring in this cohort in the future, as blood collection is minimally invasive and less painful than venepuncture. Micro-sampling could also make further pharmacokinetic, pharmacodynamics and outcome studies in the elderly more feasible.

## Introduction

Kidney transplantation is first-line treatment for younger patients with end stage renal disease as compared to dialysis, it provides both an increased life expectancy and improved quality of life (Chadban et al., 2012; Dreyer et al., 2015; Registry, 2017; Procurement and Transplantation Network, 2020; Scuderi et al., 2020). Transplantation in the elderly is becoming commonplace, with 19% of kidney transplants in Europe in 2017 (Registry, 2017), and 22% in the USA in 2019 (Procurement and Transplantation Network, 2020) performed in patients aged 65 years and older. A rise in the prevalence of transplantation in the elderly has been attributed to an aging population (Petzinger et al., 2006), increased use of expanded criteria donor kidneys (Dreyer et al., 2015), and improvements in transplant outcomes (Miura et al., 2009).

For the purposes of this review, we define elderly as 65 years of age or older (World Health Organisation, 2015). We acknowledge that this definition may seem inappropriate and outdated as patients are now transplanted well into their 70’s. The drug pharmacokinetics and pharmacodynamics of a 65 year old renal transplant recipient does not necessarily reflect a patient in their 70’s or 80’s. However, currently there are few studies reporting kidney transplant outcomes in recipients aged over 70 years which in part, may be due to the recent and rapid increase in the number of transplants in this age group.

Life-long adherence to immunosuppressant therapy is critical to support long-term graft survival and improve quality of life in the elderly. The intricate balance between under- and over-immunosuppression is increasingly complex in this cohort, due to changes in drug pharmacokinetics and pharmacodynamics (Boesmueller et al., 2011; Krenzien et al., 2015; Karpe et al., 2017). When compared to younger adults, there seems to be greater variability in drug responsiveness (Karpe et al., 2017). Presently, the elderly are the most likely age group to die with a functioning graft (Aymanns et al., 2010; Le Meur, 2015; Registry, 2017); the three main causes of death namely infection, malignancy and cardiovascular events, may be partly attributed to immunosuppressant medication usage (Turnheim, 2003; Barten et al., 2007; Le Meur, 2015; Milone, 2016).

Calcineurin inhibitors, tacrolimus and cyclosporine, are pivotal to mainstay immunosuppressant regimens in kidney transplant recipients (Chadban et al., 2012). Both agents have a narrow therapeutic window and display large pharmacokinetic variability. Therapeutic drug monitoring (TDM) is performed regularly in patients receiving these agents allowing drug doses to be adjusted to maintain drug blood concentrations within a desired therapeutic range (Chadban et al., 2012). TDM currently utilizes venepuncture sampling which requires a larger volume of blood and greater patient discomfort as some elderly patients have difficulty with venous access due to cardiovascular disease or poor venous access.

This review summarizes how possible age-related changes in calcineurin inhibitor pharmacokinetics and pharmacodynamics may influence drug dosing and monitoring in the elderly, and highlights how micro-sampling may be useful in this cohort in the future.

## Age-Related Pharmacokinetic and Pharmacodynamic Changes

Advancing biological age leads to physiological changes that can affect drug absorption, distribution, metabolism and excretion. For some drugs, there may be a slight decline in absorption due to a reduction in the surface area of the intestinal epithelium and splanchnic blood flow, a delay in gastric emptying, and an increase in gastric pH (Zaghloul et al., 1987; Staatz and Tett, 2005). However, the extent of absorption of drugs that traverse biological membranes by passive diffusion is largely unchanged in the elderly (Hutchison and O'Brien 2007) and may actually improve for basic drugs due to raised gastric pH.

Typically, the elderly have an increased fat percentage, and a reduction in lean muscle mass and total body water, compared to younger adults (Staatz et al., 2002; Boesmueller et al., 2011). Consequently, there is an increase in volume of distribution for lipophilic drugs such as the calcineurin inhibitors and a decrease for hydrophilic drugs (Staatz and Tett, 2005; Barraclough et al., 2012). Drug binding to plasma proteins may be altered in the elderly (Zaghloul et al., 1987; Falck et al., 2008). A reduction in serum albumin can cause a decrease in total drug concentration and an increase in free drug fraction, with no corresponding long-term effect on free drug concentration. This is an important consideration when interpreting TDM results for immunosuppressant drugs, as total rather than the unbound drug concentration is measured (Falck et al., 2008; Karpe et al., 2017).

Liver size and hepatic blood flow can decrease in the elderly affecting drug metabolism (Yatscoff et al., 1998; Krenzien et al., 2015). Drugs with a high liver extraction ratio, may have reduced clearance due to reduced hemoperfusion; while drugs with a low liver extraction ratio, such as calcineurin inhibitors, are often less affected as their clearance is more dependent on the activity of metabolic enzymes (Zaghloul et al., 1987). Drug metabolism via Phase-I CYP3A-mediated reactions may decline with age (Yatscoff et al., 1998; Steinebrunner et al., 2014) although the extent of any decline is often patient- and drug-specific (Steinebrunner et al., 2014). Renal excretion of drugs is frequently significantly reduced in elderly patients with the glomerular filtration rate declining by approximately 1 ml/min/1.73 m^2^ from a person’s 20’s onwards (Staatz and Tett, 2005; Barraclough et al., 2012). It is unclear if biliary excretion and enterohepatic recirculation of drugs is affected by age.

As drug clearance is generally reduced due to a decline in kidney function, and potential decline in liver function, steady-state drug concentrations may be higher in elderly patients at any given drug dose. Lipophilic drugs may be exposed to the eliminating organs of the body more slowly, as fat acts as a reservoir keeping drug out of the circulation (Falck et al., 2008), increasing the likelihood of drug accumulation with repeat dosing (Staatz and Tett, 2005). This increases the risk of toxicity {Staatz et al., 2002).

A number of membrane transporters effect drug entry into sites such as the blood brain barrier, proximal renal tubules, hepatocytes and the gut (Petzinger et al., 2006; Liang et al., 2015; Nigam, 2015; Stieger and Hagenbuch 2016). More research is required into the effects of ageing on membrane transporter expression and activity. To date studies have shown that mRNA levels of the xenobiotic-processing genes (which code for transporter expression) were significantly lower in aged mice (Miura et al., 2009) and aging reduces P-glycoprotein levels in the blood-brain barrier in healthy adults (Dambrin et al., 2000). When considering transporter effects in the elderly, the presence and impact of multiple comorbidities and concomitant medications can further complicate the situation.

Patient response to medicines is dependent on factors including homeostatic mechanisms, receptor density and affinity, and signal transduction pathways (Hämmerlein et al., 1998; Hutchison and O'Brien 2007; Corsonello et al., 2010). Generally, in the elderly there is a progressive reduction in the body’s homeostatic mechanisms, such that after a drug is administered, it takes longer for counter-regulatory mechanisms to return the body to its original state (Turnheim, 2003; Barten et al., 2007; Milone, 2016). Receptor number and responsiveness also declines with age, however, this does not necessarily change drug sensitivity or effectiveness. Instead, it can take longer for a drug to reach maximum effect, and the effect can be stronger in the elderly. Resultantly, the elderly are more likely to suffer drug side effects when dosed to the same target concentration as younger adults (Turnheim, 2003; Aymanns et al., 2010).

Immunosenescence, a reduction in the robustness of the immune system, is known to occur in the elderly (Turnheim, 2003) and is associated with a loss in the homeostatic equilibrium of the peripheral T-cell pool and a decrease in antibody response (Heinbokel et al., 2013; Le Meur, 2015). A shrinking thymus leads to a progressive decline in the naïve T-cell count and an accumulation of memory T-cells (Albright and Albright 2003; Klinger and Banasik 2015). Overabundant memory T-cells are unable to respond effectively to new immunological challenges (Albright and Albright 2003; Klinger and Banasik 2015). The increase in memory T-cells also causes a decline in naïve B-cells and a reduction in B-cell antibody response (Gibson et al., 2009; Krenzien et al., 2015; Le Meur, 2015). This leads to a reduction in the turn-over of mature B-cells which impacts both antibody specificity and the amount of plasma cells in the bone marrow (Albright and Albright 2003; Heinbokel et al., 2013). Immunosenescence impacts elderly kidney transplant patients by increasing their risk of malignancies, infections, atherosclerosis, autoimmune disorders, and neuro-degeneration disorders (Boesmueller et al., 2011; Heinbokel et al., 2013).

### Calcineurin Inhibitors

Tacrolimus and cyclosporine block calcineurin enzymatic activity preventing activated T lymphocytes from producing interleukin-2 (IL-2) (Karpe et al., 2017). By reducing IL-2 transcription, the activity of other T- and B-cells which are essential for the growth and development of the immune system are suppressed (Morris and Knechtle 2014; Milone, 2016). Blocking the calcineurin pathway also affects non-immune cells including neurons, skeletal and cardiac myoctyes leading to off-target side effects (Milone, 2016). Usage of calcineurin inhibitors is associated with a range of adverse effects (Muntean and Lucan 2013). The balance between drug effect and drug side-effects is extremely delicate, and patients may experience side-effects when dosed to a standard target concentration.

There is large variability in the pharmacokinetics of tacrolimus and cyclosporine which can be attributed to a number of factors including cytochrome P450 genotype, drug-drug interactions, patient hematocrit, patient weight, time post-transplant and patient hepatic function (Han et al., 2013; Brooks et al., 2016). Both agents have generally poor, but highly variable oral bioavailability (Han et al., 2013; Milone, 2016; Andreu et al., 2017) with absorption reduced by intestinal P-glycoprotein efflux transportation and pre-systemic metabolism by cytochrome P450 3A (CYP3A) (Andreu et al., 2017). Tacrolimus binds extensively to erythrocytes (approximately 99%) and in plasma is primarily bound to alpha-1-acid glycoprotein and albumin (Staatz and Tett 2005; Milone, 2016; Andreu et al., 2017). Cyclosporine also binds extensively to erythrocytes (Milone, 2016) and is primarily bound to lipoproteins in plasma. Once absorbed, both agents are extensively metabolized in the liver by the CYP3A system. Tacrolimus biotransformation results in generation of more than 15 metabolites, with >95% of this agent excreted by the biliary route (Staatz et al., 2002) and <1% excreted as unchanged drug in the urine and faeces (Barraclough et al., 2012). Cyclosporine biotransformation results in generation of more than 25 metabolites with >90% of this agent excreted by the biliary system, and only 6% excreted, mostly as metabolites, by the kidneys (Milone, 2016).

### Pharmacokinetics of Calcineurin Inhibitors in the Elderly

Numerous mixed effects modelling-based studies have examined the pharmacokinetics of calcineurin inhibitors in adult kidney transplant recipients with several investigating age as a covariate (Staatz et al., 2002; Han et al., 2013; Brooks et al., 2016). To date, no studies in adult recipients have reported patient age to be a significant covariate in its own right, however several studies have found that patient factors which can change with age (e.g. weight, kidney function, hematocrit), are important. Notably the sample size of elderly recipients included in each study was generally very low. Currently, four studies have specifically examined the pharmacokinetics of calcineurin inhibitors in elderly renal transplant recipients. Findings are summarized in Table 1, critique follows.

**TABLE 1:** A summary of pharmacokinetic and pharmacodynamic studies of calcineurin inhibitors in the elderly.

Study origin; Design	Agent	Sample size and age groups	Definition of “elderly”; population studied	PK/PD measure	Results/Conclusions
Falck et al. (2008) Norway; prospective pilot study	Cyclosporine	18–64 years: n = 14 >65 years: n = 11	65 + years; mean: 73 ± 3	Whole blood cyclosporine AUC_0–12h_ when stable (30–40 days post-Tx)^a^; Intracellular cyclosporine AUC_0–6h_ in T-lymphocytes^a^	Lower doses achieved same 2-h post-dose cyclosporine target concentration in elderly compared to younger adults (4.3 ± 0.8 vs. 6.1 ± 2.1 mg/day/kg; *p* = 0.025). Lower whole blood clearance (22.5 ± 5.2 vs 30.2 ± 10.4 L/h; *p* = 0.032) and intracellular clearance in elderly compared to younger adults (0.951 ± 0.32 vs 1.72 ± 0.71 × 10^6^ cells/hr; *p* = 0.0029)
Miura et al. (2009) Japan; point prevalence study	Tacrolimus	20–39 years: n = 41 40–59 years: n = 57 60–64 years: n = 12	60 + years; mean: 63 ± 3	Whole blood tacrolimus^b^ AUC_0–12h_ ^a^	Age had no impact on D/BW^d^ adjusted tacrolimus parameters
Jacobson et al. (2012) USA; longitudinal study	Tacrolimus	18–34 years: n = 348 35–64 years: n = 1831 65–84 years: n = 374	65 + years; median: 68.5	Whole blood tacrolimus trough concentration (bi-weekly weeks 1–8; bi-monthly Months 3–6)^c^	Elderly received lower doses by 1–2 mg/day and had higher troughs (129.8 vs 77.1 ng/ml.mg/kg; D/BW^d^ -adjustment) CYP3A5*1 genotype effected tacrolimus trough concentration
David-Neto et al. (2017) Brazil; longitudinal study	Tacrolimus	<60 years: n = 31 >60 years: n = 44	60 + years; mean: 65 ± 3	Whole blood tacrolimus AUC_0–12h_ (7, 30, 60, and 180 days post-Tx)^b^ ^,^ ^e^	Elderly had lower weight-adjusted doses but higher exposure (AUC - Day 7: 2286 ± 1372 vs 1369 ± 582 ng*hr*kg/mL; *p* = 0.001)
Sugiyama et al. (2008) Japan; prospective study	Cyclosporine and Tacrolimus	14–65 years: n = 29	Not defined	IC_50_ value (efficacy) measured by LIST	No correlation between log-transformed IC_50_ values and patient age. Significant correlation between age and lymphocyte % (*p* < 0.05, r = −0.475)

AUC = area-under the concentration-time curve; conc. = concentration; CYP3A5 = cytochrome P450; IC50 = drug concentration that gives 50% inhibition; LIST = Lymphocyte immunosuppressant-sensitivity testing; PK = pharmacokinetics; PD = pharmacodynamics; Tx = transplant.

^a^ AUC calculated using linear trapezoidal method.

^b^ Whole blood concentrations determined by microparticle Enzyme Immunoassay (MEIA) IMx^®^ method.

^c^ Drug concentrations measured by a validated high-performance liquid chromatography method.

^d^ D/BW = dose/ body weight.

^e^ Only 22 elderly patients performed the analysis at 60 and 180 days post-transplant.

Falck *et al* examined the pharmacokinetics of cyclosporine in a prospective, pilot study involving 11 elderly (>65 years) and 14 younger adult kidney transplant recipients (Falck et al., 2008). Elderly patients achieved the same 2-hour post-dose cyclosporine target concentration as younger adults with lower weight-adjusted doses (4.3 ± 0.8 vs. 6.1 ± 2.1 mg/kg/day; *p* = 0.025). This finding supports the idea that drug clearance decreases with advancing age. Falck *et al.* also found that elderly recipients had a higher intracellular to whole blood cyclosporine area-under-the-concentration-time curve (AUC) ratio (AUC_INTRA_/AUC_WHOLE BLOOD_ 0.035 ± 0.014 vs. 0.024 ± 0.008, *p* = 0.024), compared to younger adults, however, whole blood concentrations were similar across the cohorts (Falck et al., 2008). The authors proposed that a larger proportion of cyclosporine may be located at the site of action (within the T-lymphocyte), and suggested that drug target concentrations could be safely lowered in the elderly (Falck et al., 2008). Miura *et al* examined the pharmacokinetics of tacrolimus in 12 elderly kidney transplant recipients aged 60 years and over, by comparing their drug exposure to younger adult recipients in a point-prevalence study. Age did not influence dose- and weight-adjusted tacrolimus maximum concentration (C_max_), trough concentration (C_0_) or area-under-the-concentration-time-curve from 0 to 12 hours post-dose (AUC_0-12 hour_) (Miura et al., 2009). Jacobson *et al* conducted a retrospective, multi-centre study examining 374 elderly renal transplant recipients over the first 6 months after transplantation (Jacobson et al., 2012). They found that elderly patients (mean: 68.5 years) had higher dose-normalized tacrolimus trough concentrations than younger adults (Jacobson et al., 2012). Concentrations, normalized for both dose and body weight, were also more than 50% higher in elderly recipients compared to younger adults (Jacobson et al., 2012). A limitation to this finding is that full drug exposure was not measured, instead, only trough values were compared. However, these findings were supported by a similar study by David-Neto *et al* who also showed that elderly recipients (mean age: 65 years; *n* = 44) achieved higher target exposure (trough concentrations) and lower estimated total body clearance, with a lower normalized tacrolimus dose, compared to younger adult recipients (*n* = 31) (David-Neto et al., 2017).

With so few studies specifically designed to examine the elderly, it is still difficult to quantify the exact impact aging has on the pharmacokinetics of calcineurin inhibitors. No studies have yet compared robust elderly to more frail elderly patients. Studies to date indicate that calcineurin inhibitor doses, when lowered in elderly kidney transplant recipients, achieve the same level of immunosuppressant drug exposure as younger adults. This is possibly due to a decline in drug clearance and increased volume of distribution with advancing age.

### Pharmacodynamics of Calcineurin Inhibitors in the Elderly

*In vitro* studies have shown that calcineurin activity is inversely correlated to cytokine expression (particularly IL-2) in transplant recipients taking cyclosporine and tacrolimus (Dambrin et al., 2000). The partial inhibition observed *in vivo* may explain why there is a reduction in the responsiveness of the immune system in patients taking a calcineurin inhibitor, but not always enough to prevent graft rejection (Yatscoff et al., 1998). The relationship between tacrolimus blood concentrations and cytokine suppression in whole blood lymphocytes (Dambrin et al., 2000) is not yet reliably correlated (Steinebrunner et al., 2014). A study by Bremer and researchers showed that tacrolimus concentrations 1.5 hour post-dose resulted in strong cytokine inhibition, but cytokine suppression responses varied considerably before, and 1.5 hour after dosing, which may be attributed to different sensitivities to tacrolimus (Bremer et al., 2017). These findings suggest that there is a relationship between immunosuppressant exposure and cytokine expression, however, currently, extent of calcineurin inhibition has not been correlated to graft outcomes (Dambrin et al., 2000).

The only study to consider the effect of aging on calcineurin inhibitor pharmacodynamics was by Sugiyama *et al* *.* Age did not correlate with the calcineurin inhibitor concentrations that give 50% inhibition of peripheral-blood mononuclear cell-blastogenesis (IC_50_) values (*p* > 0.05), and hence, it was inferred that the pharmacological efficacy of immunosuppressant medicines is unlikely to be influenced by patient characteristics such as age (Sugiyama et al., 2008). This study did not examine elderly recipients (mean age 35 years ± 13; range 14–65 years), and does not fit with what is currently known about the elderly and immunosenesence.

### Transplant Outcomes in the Elderly

Current studies examining graft survival in elderly kidney transplant recipients are outlined in Table 2. Overall, the studies summarized in Table 2 show that elderly recipients are more likely to suffer graft loss than younger adults; with death with a functioning graft being the most common cause of graft loss in this group.

**TABLE 2:** A summary of studies examining graft survival in elderly renal transplant recipients.

Study Origin; Design	Sample Size	Definition of “Elderly”; Population studied	Transplant Outcomes	Conclusions
Schulak and Hricik. (1991) USA; prospective study	≥60 years: n = 29	60–69 years; mean: 62 ± 2	Graft survival in the elderly^a^ at 1 year: 84% low risk patients^b^ vs 58% high risk patients (*p* > 0.05)	Death is the major cause of graft loss in elderly recipients. Patient survival worse in high risk patients (*p* = 0.055); comorbidity screening pre-transplant important
Doyle et al. (2000) USA; retrospective study	≥60 years: n = 206 18–59 years: n = 1,640	≥60 years; mean: 64 ± 3	Graft survival^a^ at 1 year: 83% younger adults vs 73% elderly (*p* < 0.0001) graft survival^a^ at 5 years: 70% younger adults vs 56% elderly (*p* < 0.0001)	Graft loss, due to death, greater in elderly (61 vs 45%) (*p* < 0.05)
Otero-Ravina et al. (2005) Spain; retrospective study	<60 years: n = 137 >60 years: n = 484	≥60 years; mean: 64 ± 3	Graft survival^a^ at 1 year: 82% younger adults vs 73% elderly (*p* = 0.0012) and graft survival^a^ at 5 years: 70% younger adults vs 56% elderly (*p* = 0.0012) No difference in death censored graft survival at 1 and 5 years (*p* = 0.85) between groups	Survival is reduced in elderly recipients with 47% graft loss in elderly resulting in patient death (*p* < 0.0001)
Huang et al. (2009); USA retrospective study	60–69 years: n = 24,877 70–79 years: n = 6,103 80+: n = 199	≥60 years; median: 64	Graft survival^a^ at 2 years: 60–69 (89%), 70–79 (86%), 80+ (73%) (*p* < 0.0001) No difference in death censored graft survival between groups (93 vs 92% vs 91%) (*p* > 0.05)	Graft loss was greater in 80 years than 60–69 years (HR 1.78, 95% CI 1.42–2.23), but there was no difference when censored for death
Boesmueller et al. (2011); Austriaretrospective study	<70 years: n = 64 ≥70 years: n = 19	>70 years; mean: 72.7	Graft survival^a^ at 1 year: 94% younger adults vs 95% elderly (*p* > 0.05) graft survival^a^ at 5 years: 79% younger adults vs 52% elderly (*p* > 0.05) No difference in death censored graft survival at 1 and 5 years between groups (*p* = 0.51)	Primary cause of graft loss was death with a functioning graft (major reason: Cardiac failure)

HR = hazard ratio, CI = confidence interval.

^a^ Calculated by Kaplan-Meier methods.

^b^ Low risk patients = no diabetes or cardiovascular disease.

Overall, elderly kidney transplant recipients have increased risk of both morbidity and mortality, compared to younger adults (Meier-Kriesche et al., 2000; Ojo et al., 2000). Ojo and researchers reported that renal transplant recipients aged over 65 years are seven times more likely to die with a functioning graft compared to young adults (aged 18–29 years) (Impedovo et al., 2013). This finding has since been supported by Wu *et al* (Wu et al., 2005), Impedovo *et al* (Impedovo et al., 2013), Hatamizadeh *et al* (Hatamizadeh et al., 2013), and Karim et al (Karim et al., 2014), who all showed that elderly recipients had worse survival, compared to youngers adults.

This heightened mortality risk in elderly recipients may be due to infection, a well-documented side effect of calcineurin inhibitors (Lehner et al., 2015). Meier-Kriesche and researchers showed that the risk of death due to infection increases 5-fold in elderly renal transplant recipients, compared to recipients aged 30–39 years (Meier-Kriesche et al., 2000). However, mortality risk can also be attributed to other side effects such as cardiovascular disease and malignancy (Cameron, 2000; Shi et al., 2015). Furthermore, risk may be further compounded by the increased chance of recipients having multiple comorbidities (Wu et al., 2005), as well drug-drug interactions (from medicines used for these comorbidities) (Huang et al., 2009; Boesmueller et al., 2011). Consequently, from the level of current evidence, it is thought that lower calcineurin inhibitor targets in elderly recipients may reduce the side effect potential without impacting patient and graft survival, however, studies have yet to confirm this (Danovitch et al., 2007; Dreyer et al., 2015; Cossart et al., 2019).

### Therapeutic Drug Monitoring and Immunosuppressant Protocols

While TDM helps minimize problems associated with use of calcineurin inhibitors, many patients still experience rejection, infection and drug side-effects when drug exposure is in the therapeutic range (Lemaitre et al., 2015). Trough drug measurement may miss between-patient differences in drug absorption and whole blood concentrations may not fully characterize the pharmacological effect of these agents on intra-lymphocyte calcineurin activity because only unbound drug interacts with receptor sites (Wang 2007).

Current TDM methods rely on venepuncture for collection of blood samples. This process can be time-consuming, invasive and cumbersome as well as disruptive of the morning routine critical to medication adherence (Martial et al., 2016; Cossart et al., 2017). Attending venepuncture collection centers involves travel and cost for patients.

Alternative microsampling methods of blood collection based on finger-prick blood draw are currently being investigated (KDIGO, 2009). Such methods require less blood, are minimally invasive (Parker et al., 2016) and may allow patients to take blood samples at home. In dried blood spot sampling (DBS) a small volume of capillary blood is blotted onto a piece of marked filter paper and dried for future analysis (Nys et al., 2017; Edelbroek et al., 2009). In volumetric absorptive sampling (VAM) an absorbent polymeric tip is used to collect a small volume of blood by capillary action (Spooner et al., 2015; Delahaye et al., 2017; Kip et al., 2017). VAM collection has an advantage over DBS sampling in that drug concentration is measured independent of hematocrit which can influence blood viscosity and the accuracy of sample analysis (Spooner et al., 2015; Nys et al., 2017). A finger-prick sample contains capillary, arteriolar and venous blood, whereas a venepuncture sample contains only venous blood. Studies demonstrate that calcineurin inhibitor concentrations measured from finger prick samples strongly correlate with venous blood concentration (Jain et al., 1995; Webb et al., 2005; Kita and Mano 2017). New methods have been studied in other special populations i.e. paediatrics, and shown to be advantageous (Nys et al., 2017). Elderly kidney transplant recipients would be an ideal group to both test and validate microsampling protocols as these methods are likely to be beneficial – only a small amount of blood is required, minimally invasive, sampling convenience (e.g. conducted at home), and sample stability (Spooner et al., 2015; Martial et al., 2016).

Current immunosuppressant dosing and monitoring protocols make no adjustments for age (Shi et al., 2015). Elderly renal transplant recipients are predominantly excluded from clinical trials (Krenzien et al., 2015) making an evidence-based decision on immunosuppressant medicine dosing difficult (Montero et al., 2016). Furthermore, inter-patient pharmacokinetic variability increases with age due to physiological changes (Singh and Bajorek 2014), making the applicability of results from the younger adult transplant population to elderly patients more difficult. Elderly patients are also more likely to have multiple comorbidities resulting in polypharmacy and drug-drug interactions (Akhtar and Ramani, 2015), which only further highlights the importance of achieving drug doses within the target range.

Only one study has examined the impact of an altered calcineurin dosing protocol on graft outcomes in older adults. Badowski *et al* lowered the tacrolimus dosing targets in a cohort of 88 patients aged over 60 years (group 1 target concentration: 10–12 ng/ml; group 2 target concentration: 8–10 ng/ml) and found that the reduction in tacrolimus target drug concentration improved graft survival, without increasing the risk of acute rejection (*p* = 0.006) (Badowski et al., 2009). Patients were followed-up for approximately 2 years post-transplant (group 1: 23.8 ± 14.2 months; group 2: 21.3 ± 11.8 months). Further studies are required to quantify how lowered immunosuppressant doses or target concentrations may support graft and patient survival and potentially reduce the development of drug side effects in elderly and frail elderly transplant recipients.

## Discussion/Conclusion

The intricate balance between under- and over-immunosuppression becomes increasingly more complex in elderly transplant recipients, due to changes in pharmacokinetics and pharmacodynamics. Overall findings suggest that calcineurin inhibitor doses may need to be lowered in elderly recipients. However, only one study to date has examined the impact of aging on effectiveness of calcineurin inhibitor activity. Immunosenescence is known to occur in the elderly and likely impacts elderly kidney transplant patients by increasing their risk of malignancies, infections, artherosclerosis, autoimmune disorders, and neuro-degeneration disorders. However, the evidence around age-related pharmacology, particularly in immunosuppression, is sparse. More studies are needed to link drug exposure to outcomes in this cohort. There is also a need to validate micro-sampling for TDM of calcineurin inhibitors in the elderly as micro-sampling has shown to be promising in other special populations. In the future, micro-sampling based TDM may better support monitoring of drug exposure and long-term patient outcomes in the elderly and allow for further pharmacokinetic and pharmacokinetic studies in this cohort.
